# Prevalence of syphilis and associated factors among pregnant women in Brazil: systematic review and meta-analysis

**DOI:** 10.61622/rbgo/2024rbgo28

**Published:** 2024-05-27

**Authors:** Iana Mundim de Oliveira, Regilane Cordeiro dos Santos, Renan Alves Silva, Rosane Ribeiro Figueiredo Alves, Bruno César Teodoro Martins, Leonardo Ribeiro Soares

**Affiliations:** 1 Universidade Federal de Goiás Goiânia GO Brazil Universidade Federal de Goiás, Goiânia, GO, Brazil.; 2 Universidade Federal de Campina Grande Cajazeiras PB Brazil Universidade Federal de Campina Grande, Cajazeiras, PB, Brazil.

**Keywords:** Syphilis, Pregnant women, Pregnancy complications, infectious, Pregnancy, Prevalence, Brazil

## Abstract

**Objective:**

This systematic review accompanied by a meta-analysis aimed to estimate the prevalence of syphilis in pregnant women in Brazil and describe its associated factors.

**Methods:**

Following the establishment the search strategies and the registration of the review protocol in PROSPERO, we conducted a search for relevant articles in the Pubmed, LILACS, Science Direct, SciELO and Web of Science databases. Our inclusion criteria were cross-sectional studies published between 2005 and 2023, with no language restrictions. The combined prevalence of syphilis infection was estimated using the random effects model in the R Software with a 95% confidence interval (95% CI) and p < 0.01 as statistically significant.

**Results:**

A total of 24 articles were recruited, which together investigated 221,884 women. The combined prevalence of syphilis in pregnant women in Brazil was 1.79% (95% CI: 1.24-2.57%), and the main factors associated with its occurrence were black and brown skin color, low education and factors related to the partner.

**Conclusion:**

There was a high prevalence of syphilis in pregnancy in Brazil, mainly associated with socioeconomic factors.

## Introduction

Syphilis is considered a serious public health problem. Although there are preventive measures and accessible and effective treatment options, its prevalence is increasing worldwide, bringing several consequences for health, especially during the gestational period.^(1)^ Infection during pregnancy, when not detected early or not treated properly, poses a great risk to the fetus. Recent data reveals a pooled prevalence of syphilis infection among pregnant women of 0.8% (CI: 0.7-0.9%), getting at 3.3% (CI: 2.2-4.6%) in low income countries.^(2)^ In Brazil, according to the latest Syphilis Epidemiological Bulletin, from 2005 to june 2022, 535,034 cases of syphilis among pregnant women were notified in the System for Notification of Diseases (SINAN). The southeast region stands out with the highest number of cases corresponding to more than 40%.^(3)^ It is estimated that up to 80% of cases of syphilis during pregnancy are diagnosed late, in the second or third trimester of pregnancy,^(4)^ and the proportion of cases treated appropriately, that is, with the use of penicillin in a dose corresponding to the stage of the disease and completed within 30 days before childbirth, is less than 70% in the country.

Among the factors that contribute to the high prevalence of syphilis in Brazil, sociodemographic characteristics like black and brown skin color, low socioeconomic status, low education and, above all, failure in prenatal care can be observed.^(4)^ In Brazil, the population most affected by the disease are brown and young women, aged between 20 and 29 years, representing 48.6% of all reported cases of syphilis in pregnant women. In this same group, there is also a report of lower adherence to the recommended number of consultations during prenatal care.^(5,6)^

The mandatory registration of cases of Gestational Syphilis (GS) on SINAN has been established in Brazil since 2005.^(7)^ The inclusion of GS as a notifiable disease in SINAN is justified by its high prevalence and vertical transmission rate, which can range from 30% to 100% if untreated or inadequately treated.^(7)^ However, flaws in the reporting system, in the quality and provision of prenatal care constitute barriers to controlling this disease.^(8,9)^

It is believed that knowledge about the prevalence of syphilis during pregnancy in the country and its possible associated factors, based on studies with primary data, can provide subsidies for the establishment of public health policies aimed at controlling and preventing the disease, in order to interrupting its chain of transmission and preventing negative outcomes and fetal deaths resulting from congenital syphilis. Thus, this study aimed to estimate the prevalence of syphilis among pregnant women in Brazil and describe its associated factors.

## Methods

This systematic review and meta-analysis was developed based on the *Preferred Reporting Items for Systematic Reviews and Meta-Analyses* (PRISMA),^(10)^ having previously been registered in the *International Prospective Register of Systematic Reviews* (PROSPERO) under the number CRD42019120263.

### Search Strategies

The source was conducted in october 2023. Scientific articles published in journals and available in six electronic databases through advanced search. Were used: *National Library of Medicine* (PubMed), *Latin American Literature on Health Sciences* (LILACS), *Science Direct, Scientific Electronic Library Online* (SciELO), *Scopus*, *Web of Science* and Portal Capes, using keywords included in the *Medical Subject Headings* (MeSH terms) and *Health Sciences Descriptors* (DeCs) combined by boolean operator AND:

**Table:** 

#1: Syphilis
#2: Pregnancy [OR] pregnant women
#3: Epidemiology [OR] prevalence [OR] cross sectional studies
#4: Brazil
#5: #1 [AND] #2 [AND] #3 [AND] #4

In addition, references were also consulted to check whether studies not found in the databases. All retrieved studies were exported to the Rayyan application for deduplication and title, abstract and full-text screening by three independent researchers.

### Eligibility criteria

The inclusion criteria adopted were: cross-sectional studies available in full, carried out in Brazil, reporting the prevalence of syphilis and/or possible associated factors and published in any language in the period between 2005 and 2023. The choice of this period is due to the beginning of the syphilis in pregnant women's notification in Brazil, representing a milestone in the epidemiology of syphilis. We excluded incomplete articles, conference proceedings, book chapters, case reports, literature reviews and studies that reported only the prevalence of syphilis acquired and congenital, excluding the gestational form.

### Study selection and data extraction

The titles and abstracts were read by three of the authors (IMO, BCTM and RCS), independently, to verify possible duplicate studies, as well as to meet the pre-established inclusion criteria. After an initial selection, eligible studies were read in full by the authors to compose the sample. Cases of disagreement between researchers were resolved by another researcher (RAS). Also independently, a predefined form was used by the two researchers to extract the following data from each study: name of the main author and year of publication, study location/region, sample size, test methods for detecting syphilis and its prevalence, as well as the methodological quality of the studies.

### Methodological quality assessment

To assess the quality of each selected study, the Critical Appraisal Checklist for Studies Reporting Prevalence Data, developed by the Joanna Briggs Institute (JBI) was used.^(11)^ The instrument assesses the presence of the following quality indicators: (1) Was the sample appropriate to address the target population? (2) Were study participants sampled in an appropriate way? (3) Was the sample size adequate? (4) Were the study subjects and setting described in detail? (5) Was the data analysis conducted with sufficient coverage of the identified sample? (6) Were valid methods used to identify the condition? (7) Was the condition measured in a standard and reliable way for all participants? (8) Was there appropriate statistical analysis? (9) The response rate was adequate. Responses were scored 0 for "Not appropriate and or not reported" and 1 for "Yes" response. The total scores of the studies varied between 0 and 9.

### Data analysis

The extracted data were inserted into a table in Microsoft Excel (version 2010) and exported to Software R (version 3.5.1) for statistical analysis. The forest plot was used to illustrate the results of the meta-analysis. To estimate the pooled prevalence of syphilis, the random effects model was used with a 95% confidence interval (95%CI). Statistical heterogeneity was assessed using Cochran's Q test and its degree was measured with I^2^ in whichvalues above 75% indicate considerable heterogeneity.^(12)^ The publication bias was investigating using visual inspection of a funnelplot and Egger test.^(13)^ Statistical significance was set at p<0.01. The factors associated with the prevalence of syphilis in pregnant women extracted from the studies were presented descriptively.

## Results

**Figure 1:**
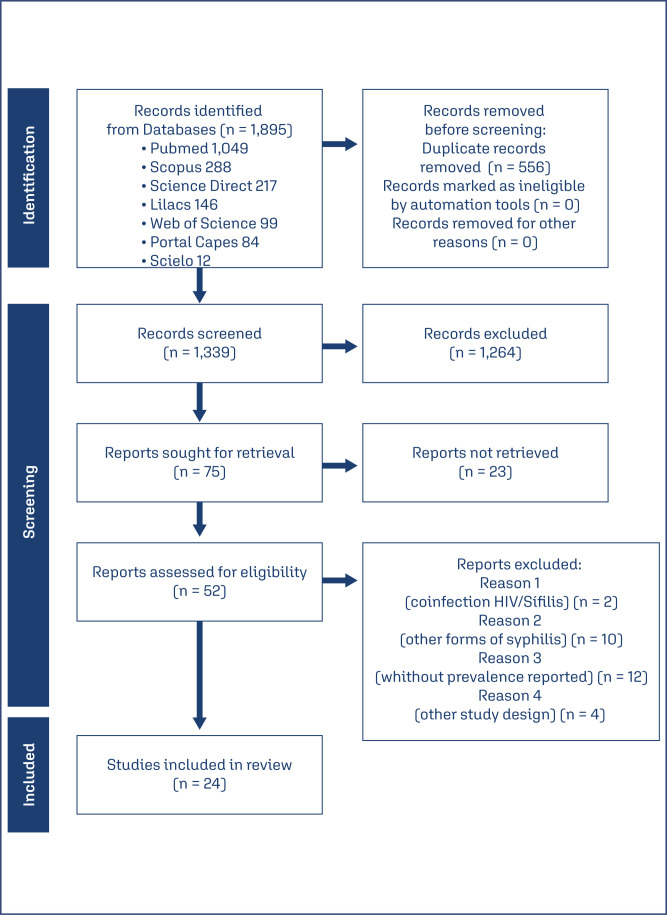
Study selection process.

A total of 1,895 studies were retrieved from the databases: 1,049 from PubMed, 288 from Scopus, 217 from Science Direct, 146 from LILACS, 99 from Web of Science, 84 for Portal Capes and 12 from SciELO. After excluding duplicates and screening titles and abstracts, 75 studies were selected for full-text reading. Of these, 24 cross-sectional studies including 221,884 pregnant women met the eligibility criteria. The study selection process is illustrated in figure 1.

The studies were carried out between 2007 and 2020 and the population ranged from 185 to 54,813 pregnant women, as shown in chart 1. Most studies used the combination of non-treponemic and treponemic test as a diagnostic method (n=15). The most used test was the Veneral Disease Research Laboratory (VDRL), which appeared in 20 of the 25 studies included in this review (Chart 1).

**Chart 1:** General characteristics of the studies included in the systematic review and meta-analysis of the prevalence of syphilis in pregnant women in Brazil

Author and year of publication	Region, location	Sample of pregnant women	Average age	Study design	Diagnostic method	Prevalence (%)	Study quality score by JBI Appraisal Checklist (1 to 9)^(11)^
Szwarcwald et al. (2007)^(14)^	Brazil	13, 267	-	Cross-sectional	VDRL	1.10% (146)	6
Inagaki et al. (2009)^(15)^	Aracaju, SE	9,550	24.5	Cross-sectional	VDRL and DBS	0.90% (n = 86)	8
Miranda et al. (2009)^(16)^	Vitória, ES	1,380	24.2	Cross-sectional	TR and VDRL	0.43% (n = 14)	8
Gonçalves et al. (2010)^(17)^	São José do Rio Preto, SP	561	27.2	Cross-sectional	VDRL	0.89% (n = 10)	5
Machado Filho et al. (2010)^(18)^	Amazônia ocidental, AM	674	23.9	Cross-sectional	VDRL and chemiluminescence assay	1.04 % (n = 8)	9
Araújo et al. (2013)^(19)^	Fortaleza, CE	222	-	Cross-sectional	VDRL	7.66% (n = 17)	8
Domingues et al. (2013)^(20)^	Rio de Janeiro, RJ	2,422	-	Cross-sectional	VDRL	1.90% (n = 46)	9
Nóbrega et al. (2013)^(21)^	Salvador, BA	3,300	25.8	Cross-sectional	VDRL and Trepanostika TP	0.52% (n = 28)	7
Pires et al. (2013)^(22)^	Vitória da Conquista, BA	2,232	-	Cross-sectional	VDRL and ELISA	2.24% (n = 50)	6
Boa-Sorte et al. (2014)^(23)^	Salvador, BA	692	27.1	Cross-sectional	DBS and ELISA	0.72% (n = 5)	9
Fernandes et al. (2014)^(24)^	Marabá, PA	477	-	Cross-sectional	VDRL	1.89% (n = 9)	8
Cunha and Merchan-Hamann (2015)^(25)^	Brazil	36,713	25.2	Cross-sectional	TR, VDRL, FTA-Abs and TPHA	0.89% (n = 327)	9
Moura et al. (2015)^(26)^	Maceió, AL	54,813	23.3	Cross-sectional	VDRL and DBS	2.80% (n = 1535)	8
Benzaken et al. (2017)^(27)^	Amazonas, AM and Roraima, RR	4,144	22.5	Cross-sectional	TR	1.52% (n = 63)	8
Bernardi et al. (2017)^(28)^	Francisco Beltrão, PR	185	-	Cross-sectional	VDRL, RPR, FTA-Abs and TR	6.49% (n = 12)	6
Soares et al. (2017)^(29)^	Guarapuava, PR	2,868	-	Cross-sectional	VDRL	1.39% (40)	6
Padovani et al. (2018)^(30)^	Paraná, PR	53,684	-	Cross-sectional	VDRL and FTA-Abs	0.57% (n = 306)	7
Silva et al. (2020)^(31)^	Paraná, PR	25,463	-	Cross-sectional	VDRL	0.97% (n = 247)	7
Almeida et al. (2021)^(33)^	Campos dos Goytacazes, RJ	970	(27.9 ± 6.65)	Cross-sectional	VDRL	2.68% (n = 26)	7
Roehrs et al. (2021)^(32)^	Florianópolis, SC	4443	-	Cross-sectional	Treponemic and non-treponemical test	4.77% (n = 212)	6
Yeganeh et al. (2021)^(34)^	Porto Alegre, RS	400	-	Cross-sectional	VDRL and rapid test	10.50% (n = 42)	8
Reis et al. (2022)^(35)^	Ribeirão Preto, SP	2675	-	Cross-sectional	Treponemic and non-treponemic test	2.69% (n = 72)	9
Scherer et al. (2022)^(36)^	Pelotas, RS	350	27.8± 6.5	Cross-sectional	Rapid test	2.00% (n = 7)	7
Guedes et al. (2023)^(37)^	Juiz de Fora, MG	399	25±5.8	Cross-sectional	Treponemic and non-treponemic test	9.02% (n = 36)	8

VDRL - venereal disease research laboratory; RT - rapid test, RPR=rapid plasma regain; FTA-Abs - fluorescent treponemal antibody absorption test, TPHA - treponema pallidum hema gglutination test, DBS - dried blood spot; ELISA - enzyme linked immunosorbent assay

**Figure 2:**
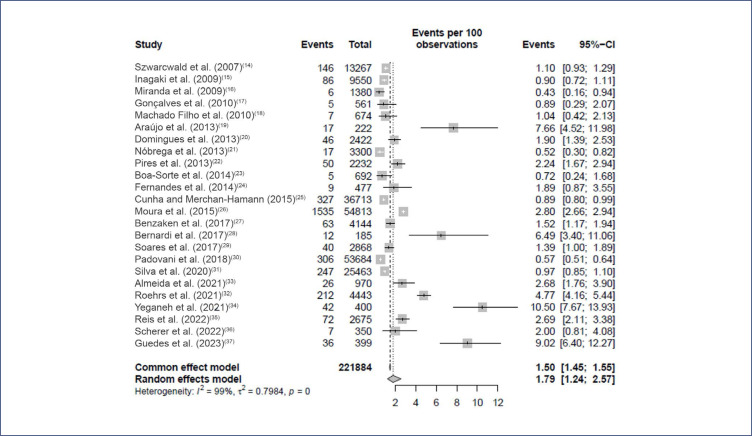
The combined overall prevalence of syphilis among pregnants in Brazil

About the quality of studies, as shown in chart 1, most of the studies scored 8 on the JBI instrument (33%),^(15,16,19,24,26,27,34,37)^ and only one of the studies scored 5^(17)^ (Chart 1). The combined overall prevalence of syphilis among pregnant women in Brazil according to the random effects model was 1.79% (95% CI: 1.24-2.57 %) with high heterogeneity observed between studies (I^2^ = 99% and τ = 0.7984, p = 0) (Figure 2).

Most studies (n=14) reported the factors associated with the occurrence of syphilis in pregnant women in Brazil, the most discussed among the studies were black and brown skin color, low level of education, and factors related to the partner like conflictual relationship, drug use and previous history of STIs by the partner (Chart 2).

**Chart 2:** Main risk factors/associated with syphilis in pregnant women in Brazil

Risk factors	Proportion of studies (%)
Black and brown skin color^(20,24–26,30,34)^	n = 6(42.8)
Low education^(19,20,24–26,30)^	n = 6(42.8)
Factors related to the partner (e.g. conflictual relationship, drug use and previous history of STIs by the partner)^(19,21,34,35,37)^	n = 5(35.7)
Factors related to pregnancy and prenatal care (e.g. late start of prenatal care, low number of consultations)^(20,24,26,30)^	n = 4(2.5)
Low income^(21,22,25,30)^	n = 4(28.5)
Use of alcohol and illicit drugs^(16,19)^	n = 2(14.3)

**Figure 3:**
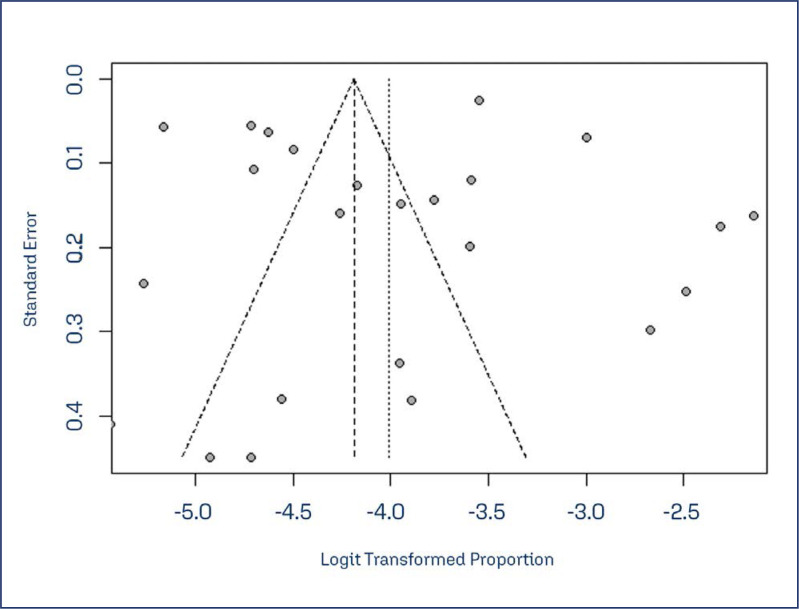
Funnel plot of the studies included in the systematic review

Other factors mentioned were age, specially under 30 years old, previous history of STI or violence and early sexual intercourse. The publication bias was assessment through visual inspection of the funnel plot, which appears symmetrical, indicating low risk of bias. The Egger test was t = −0.40 (p = 0.69) (Figure 3).

Sensitivity analyzes were performed to assess the influence of study quality as well as population size on the meta-analysis. However, when studies with a score lower than 8 in the quality assessment or with a population of less than a thousand women were excluded, there was no reduction in heterogeneity (analyses available in the Supplementary Material 1).

## Discussion

This study presents the combined prevalence of syphilis in pregnant women in Brazil between 2005 and 2023. Although similar publications already exist, this review specifically addresses Brazil and updates a previous publication about this relevant topic. According to the results obtained, syphilis during pregnancy presents itself as a public health problem, especially affecting socially less favored classes.

The estimated pooled prevalence of syphilis in the present meta-analysis was 1.79% (95% CI: 1.24-2.57%) among pregnant women in Brazil. This prevalence was similar to results found in a previous primary study conducted in the five regions of the country with 23,894 pregnant women, which showed a prevalence of 1.02%.^(20)^ However, it was lower than the prevalence obtained by a systematic review carried out with 15 studies in Latin American countries, including Brazil, which was 2.6% (95% CI: 1.2–3.9%).^(20,38)^ This difference is probably due to the influence of socioeconomic and sociocultural heterogeneity among Latin American countries.

Corroborating this hypothesis, when compared to studies carried out in the African continent, this study demonstrates a low prevalence of syphilis during pregnancy in Brazil. In Ethiopia, a cross-sectional study including 210 pregnant women reported a prevalence of 1.9% (95%CI: 0.5-3.5%)^(39)^ Another systematic review study with meta-analysis carried out in sub-Saharan Africa between 1999 and 2018 found a syphilis prevalence of 2.9% among pregnant women (95%CI: 2.4%-3.4%).^(40)^

The two most comprehensive cross-sectional studies included in this review found similar prevalences (0.89% and 1.10%) with the estimate obtained in our meta-analysis (1%).^(14,25)^ However, this prevalence was considerably lower when compared to local findings as described by Araújo et al. (2013)^(19)^ in Fortaleza – Ceará (CE), and Bernardi et al. (2017)^(28)^ in Francisco Beltrão – Paraná (PR), whose prevalence rates were, respectively, the most expressive among all evaluated (7.70% and 6.49%). It is believed that such differences derive mainly from the expansion of health care networks, with the reorganization of primary care in some locations, from the use of distinct research methodologies between studies and, above all, from regional differences in the detection rate. of syphilis in the prenatal period.^(3,14,19,25,28)^

Regarding the factors associated with the occurrence of syphilis in pregnant women, socioeconomic factors stand out. The results of this systematic review indicate that black and brown color skin, low education, factors related to the partner like conflictual relationship, drug use and previous history of STIs by the partner and factors related to pregnancy and prenatal care in addition to unfavorable living conditions, significantly influence the rates of syphilis infection during the gestation. A study carried out in Fortaleza – Ceará (CE) reported maternal characteristics similar to those found in this research, corroborating the influence of socioeconomic conditions in the determination of gestational syphilis.^(20)^ Therefore, it is believed that knowledge of these factors can contribute to the formulation of specific public policies for the population most in need.

As described in the literature, two phenomena corroborate for women with low education and low income to lead the prevalence rates of syphilis in pregnancy, namely, the limited understanding about the importance of measures to prevent sexually transmitted infections (STIs),^(41,42)^ and the low adherence to prenatal care,^(6)^ resulting in the failure to carry out the tests recommended by the Ministry of Health, including the diagnostic test for syphilis. In a study carried out in the city of Rio Grande – Rio Grande do Sul (RS), the prevalence of non-performing serology for syphilis was 2.9%, with a higher proportion in mothers of black color, low education and low income.^(43)^

The age group most affected by syphilis during pregnancy in brazilian women was between 20 and 29 years old. Similar findings were described in places like Macaé - Rio de Janeiro (RJ) and Americana - São Paulo (SP), based on secondary data provided by the Municipal Epidemiological Surveillance.^(1,44)^ It is considered that in this age group there is a greater propensity to adopt risky sexual behaviors, such as multiple partners, favoring the transmission of the infection.^(45)^

Late initiation of prenatal care is also a factor with a strong impact on the detection and treatment of syphilis. The Ministry of Health recommends that all pregnant women should be tested for syphilis, at least, in the first prenatal consultation, at the beginning of the third trimester and during hospitalization for childbirth, since this diagnosis is basically serological.^(46)^ The *Venereal Disease Research Laboratory* (VDRL) test is the most used for detection, monitoring of response and cure control of the disease.^(46,47)^ It is a highly sensitive test, but with low specificity.^(47,48)^ Therefore, whenever possible, pregnant women should perform a treponemal test in combination, such as direct immunofluorescence (FTA-ABS), hemagglutination test (TPHA) or immunoenzymatic assay (ELISA), in order to increase the positive predictive value of a reagent result.^(47)^ Eighteen of the twenty-five studies selected for this review used the VDRL as a diagnostic method,^(14–22,24,26,28–32,33,34,36)^ however, eight of these used only it.^(14,17,19,20,24,29,31,32)^ It is believed that this fact may also have influenced the heterogeneity of estimates of syphilis during pregnancy between studies.

This study has limitations, such as the high heterogeneity between studies. Due to the great spatial and methodological variability of the studies, pooled estimates were also calculated for specific characteristics, like studies with population size > 1000 women and score > 8 in the JBI instrument (supplementary material). However, sensitivity analyzes were not able to explain the higher heterogeneity, but we carefully explored the available literature in search of factors that could explain such heterogeneous results. In addition, the selection of studies, data extraction and assessment of the quality of the included articles can be influenced by judgments on the part of the authors, and to mitigate this risk, such steps were performed by more than one researcher, independently. Future studies can be conducted for to investigate the particularities between regional subgroups and sociodemographic characteristics regarding the prevalence of syphilis in the pregnant population.

## Conclusion

There was a high prevalence of syphilis in pregnancy in Brasil, mainly associated with socioeconomic factors, like color skin and scolarship, factors related to the partner and low adherence to prenatal care.

## Figures and Tables

**Figure:**
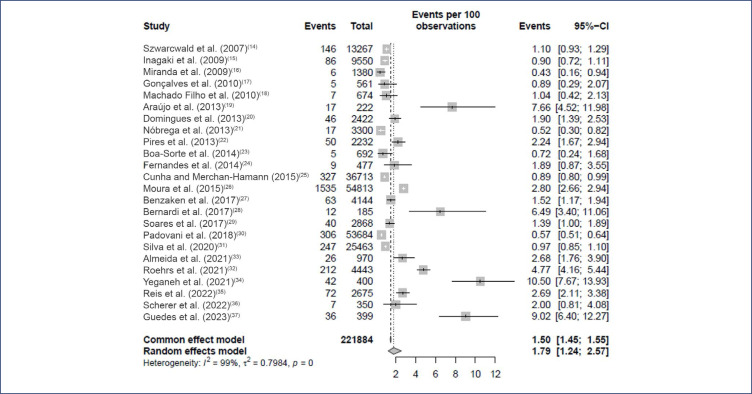
Metanalysis with all studies

**Figure:**
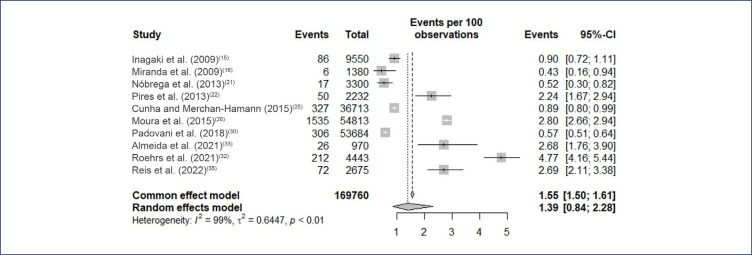
Funnel plot of all the included studies – publication bias

Subgroup: studies with population > 1000 and that used treponemal AND nontreponemal tests

**Figure:**
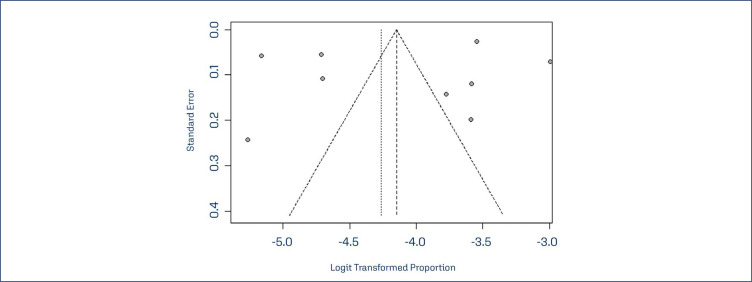
Funnel plot of "item c"

**Figure:**
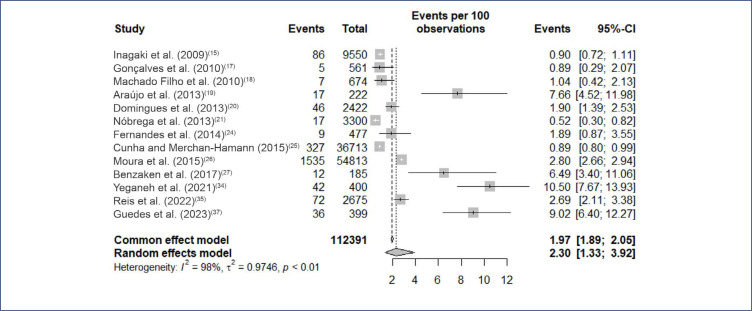
Sensibility analysis

Subgroup: studies with score >=8 in the JBI

**Figure:**
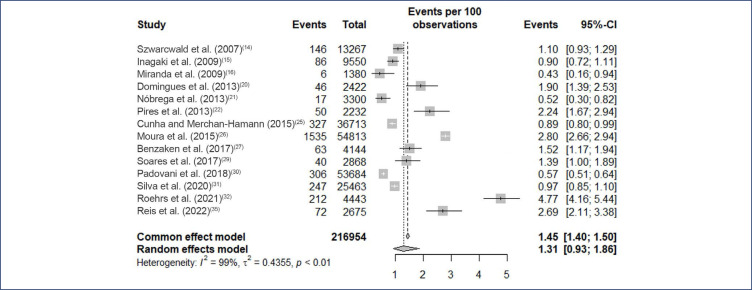
Sensibility analysis

Subgroup: studies with population > 1000
